# Oral microbiota in cesarean-delivered puppies

**DOI:** 10.3389/fvets.2025.1711728

**Published:** 2025-12-08

**Authors:** Matteo Burgio, Francesco Pellegrini, Lorenza Frattina, Alice Carbonari, Giovanni Bramante, Olga Maria Andriulo, Michele Camero, Gianvito Lanave, Antonio Parisi, Vito Martella, Annalisa Rizzo, Vincenzo Cicirelli

**Affiliations:** 1 Department of Veterinary Medicine, University of Bari Aldo Moro, Valenzano, Italy; 2 Istituto Zooprofilattico di Puglia e Basilicata, Putignano, Italy; 3 Department of Pharmacology and Toxicology, University of Veterinary Medicine, Budapest, Hungary

**Keywords:** oral microbiome, dogs, c-section, mother, puppies

## Abstract

**Introduction:**

The microbiota plays a fundamental role in host health, and alterations in its composition have been associated with numerous pathological conditions. The neonatal period is a critical window for establishing a stable microbiota that shapes long-term health. The aim of this study was to characterize the oral microbiota of cesarean-delivered puppies at birth and 15 days postpartum, using 16S rRNA gene sequencing. This microbiota was compared with the maternal oral and colostrum microbiota.

**Methods:**

The study included 15 puppies delivered by cesarean section from four French Bulldogs. Oral swabs were collected from puppies at birth (T0) and day 15 (T15), and from dams together with colostrum before anesthesia. DNA was extracted and the full-length 16S rRNA gene amplified with universal primers. Libraries were prepared, purified, and sequenced on a MinION Mk1C for 24 h. FastQ files were analyzed with EPI2ME (Fastq 16S), and taxonomic assignment was performed using the NCBI_16S database via BLAST.

**Results:**

Microbial DNA was detected in neonatal samples at birth, indicating that colonization had already begun. Diversity analyses showed significant differences between the puppies’ oral microbiota at T0 and T15 (*p* = 0.006), as well as between neonates at T0 and their mothers (*p* = 0.018). By contrast, no significant differences in alpha diversity were observed between puppies at T15 and their mothers, suggesting convergence toward an adult-like microbial profile. Colostrum did not show significant differences compared with the puppies’ oral microbiota at both time points, suggesting it may act as a possible, though not exclusive, source of microbial transfer.

**Conclusion:**

The oral microbiota of cesarean-delivered puppies undergoes rapid compositional changes within the first 15 days of life, marked by increased alpha diversity and a shift toward a microbial profile resembling that of the mother. Initial colonization likely derives from non-oral maternal or environmental sources, with convergence by day 15 due to maternal contact. Maternal colostrum did not significantly influence oral diversity, though it may act as a vector of microbial transfer. These findings underscore the dynamic nature of early-life colonization and contribute to our understanding of host–microbiota interactions in a One Health context.

## 1 Introduction

The term microbiota describes the assemblage of microorganisms that colonize specific anatomical and ecological niches of a multicellular host, forming complex, dynamic communities that play essential roles in maintaining homeostasis (1, 2). These microbial consortia can reside on mucosal surfaces such as the gastrointestinal, respiratory, urogenital tract as well as on cutaneous and oral epithelia, establishing symbiotic, commensal, or pathogenic relationships with the host (3). The study of microbiota has grown in scientific attention in recent years, driven by the advent of next-generation sequencing (NGS), metagenomics, and other high-throughput culture-independent methodologies (4). These tools have enabled a paradigm shift in microbial ecology, facilitating in-depth exploration of community structure, diversity, functional potential, and host–microbe interactions. Alterations in the microbiota, a condition known as dysbiosis, have been implicated in the pathogenesis of numerous disorders, including inflammatory bowel disease, obesity, diabetes mellitus, atopic dermatitis, autoimmune syndromes, and even behavioral abnormalities (5–8). Given these findings, the accurate characterization and functional assessment of microbiota in specific anatomical compartments have become critical for distinguishing between resident commensals and opportunistic pathogens, as their relative abundance, spatial distribution, and temporal dynamics may significantly influence health outcomes. One of the most insightful frontiers in microbiota research pertains to the ontogeny of microbial colonization during the perinatal and early postnatal periods. It is now widely accepted that early microbial exposures are not only instrumental in shaping the neonatal immune landscape but also have long-lasting effects on metabolic programming and disease susceptibility (9). Traditionally, the intrauterine environment was considered sterile, and colonization was thought to commence at parturition. However, emerging evidence from human and animal studies has challenged this established belief, suggesting that microbes can be transmitted from maternal sources during pregnancy, including the oral cavity, placenta, and amniotic fluid, although this hypothesis remains controversial and subject to ongoing debate (10, 11). In this context, the oral cavity constitutes a particularly relevant yet understudied site. It acts as a gateway between the external environment and internal compartments and may serve as an initial colonization niche immediately following birth (12). In veterinary medicine, studies investigating the early-life microbiota have predominantly focused on the gastrointestinal tract, with limited attention to other anatomical regions such as the oral cavity (5, 13). A notable exception is a study conducted in Slovakia in 2021, which attempted to characterize the oral microbiota of newborn puppies immediately post-partum, comparing those delivered vaginally with those delivered by cesarean section. However, that study relied exclusively on culturomic approaches, which, while valuable, are inherently restricted to the subset of microorganisms that are viable and cultivable under standard laboratory conditions (14). Such methods inevitably fail to capture the full spectrum of microbial diversity, particularly fastidious or uncultivable taxa that may play critical roles in early colonization and immune modulation (15). The present study aimed to address these gaps by employing high-resolution, culture-independent molecular techniques to investigate the composition and early temporal dynamics of the oral microbiota in newborn puppies. Specifically, we assessed the temporal variation of the oral microbiota in cesarean-delivered puppies, comparing day 0 with day 15 of life, trying to correlate with potential maternal sources (oral and colostral microbiota).

## 2 Materials and methods

### 2.1 Ethical statements

All procedures were conducted in accordance with institutional guidelines for the welfare and use of live animals, with the informed consent of the owners and the approval of the Ethics Committee of the University of Bari “Aldo Moro” on 27/03/2023 (protocol no. 8/2023).

### 2.2 Animals

This study was carried out at the Department of Veterinary Medicine, University of Bari “Aldo Moro” from July to September 2023 and involved the enrollment of 4 French Bulldog breeding females (Dams A, B, C, D). All dams came from the same breeding facility and were raised under uniform management and feeding conditions. To ensure their health status, the dams underwent clinical examination, reproductive ultrasound, hematobiochemical testing, and evaluation of serum progesterone (P4) levels. The dams had an average age of 3 years (range: 2 years and 7 months – 3 years and 4 months), with a Body Condition Score (BCS) between 2.5 and 3, and an average body weight of 12.5 kg (range: 11.7–13.1 kg) (Table 1). All dams were artificially inseminated with fresh semen, and pregnancy was confirmed by ultrasound 21 days post-insemination. The pregnancies were monitored through regular check-ups, ultrasounds, and P4 assessments up to the time of the scheduled cesarean section (16). No antimicrobials, drugs, or supplements were administered to the dams from the time of enrollment until the completion of sampling. A total of 15 puppies were delivered. For each puppy, a medical record was compiled, including sex, APGAR score, body weight, temperature, and any presence of congenital malformations.

**Table 1:** Data of the dams (ID, age, body weight, BCS, number of puppies born) and their respective puppies (ID code, sex, birth weight).

Dams data		Puppy’s ID	Sex	Weight (Kg)
A 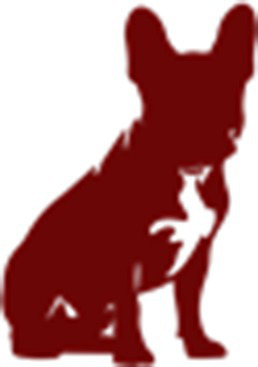	Age: 3 y and 2 m Weight: 13.1 Kg BCS: 3 N° puppies: 3	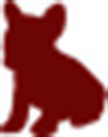 A1	F	0.29
		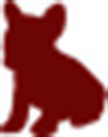 A2	M	0.25
		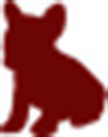 A3	M	0.22
B 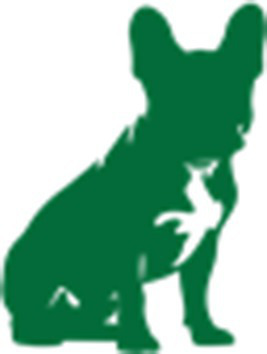	Age: 2 y and 7 m Weight: 12.2 Kg BCS: 2.5 N° puppies: 5	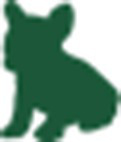 B1	F	0.27
		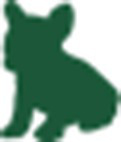 B2	F	0.2
		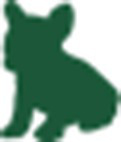 B3	M	0.26
		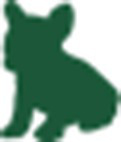 B4	F	0.18
		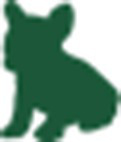 B5	F	0.34
C 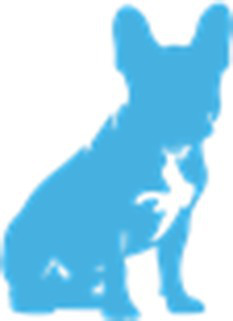	Age: 3 y and 4 m Weight: 12.8 Kg BCS: 3 N° puppies: 4	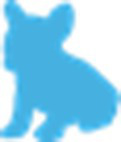 C1	F	0.32
		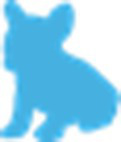 C2	M	0.3
		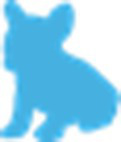 C3	F	0.29
		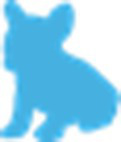 C4	M	0.31
D 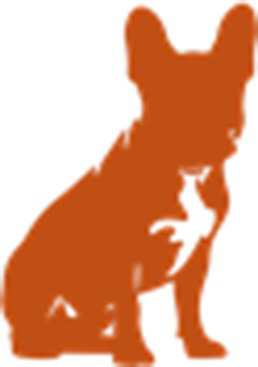	Age: 3 y and 1 m Weight: 11.7 Kg BCS: 2.5 N° puppies: 3	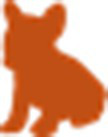 D1	M	0.28
		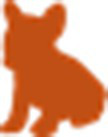 D2	F	0.26
		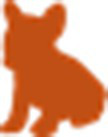 D2	F	0.22

### 2.3 Sample collection

Twenty minutes before surgery, oral swabs were collected from the dams using sterile swabs (Cliniswab, Transport Swabs, APTACA S.p. A., Canelli, Asti, Italy). Swabbing was performed by rotating the swab for 5 s over the oral mucosa. A colostrum aliquot was also collected before trichotomy and surgical scrubbing into 2 mL sterile Eppendorf tubes, sampling secretion from at least three different nipples. Immediately after birth, oral swabs were collected from each puppy using sterile swabs (Cliniswab, Transport Swabs, APTACA S.p. A., Canelli, Asti, Italy). The swabs were inserted sterilely into the oral cavity right after the rupture and removal of fetal membranes and before neonatal resuscitation procedures. At 15 days of age, oral swabs were collected again from each puppy following the same procedure. All sampling procedures were performed by the same operator, wearing sterile gloves to ensure consistency and minimize the risk of contamination. All samples were transported at +4 °C to the Infectious Diseases Laboratory at the Department of Veterinary Medicine, University of Bari, and processed immediately upon arrival.

### 2.4 Laboratory analyses

#### 2.4.1 Nucleic acid extraction, amplification, and 16S rRNA gene sequencing

Oral swabs were homogenized in 2 mL Eppendorf tubes containing Dulbecco’s Minimal Essential Medium (DMEM) and mixed using a Tissue Lyser (Qiagen GmbH, Hilden, Germany) at 30 Hz for 5 min. Homogenized samples were centrifuged at 10,000 × g for 3 min. Then, 200 μL of the supernatant was used for DNA extraction using the DNeasy PowerSoil PRO kit (Qiagen S.p. A., Milan, Italy) according to the manufacturer’s instructions. ‘DNA extracts were subjected to PCR to amplify the full-length (1,500 bp) 16S rRNA gene using universal primers listed in Table 2 and the TaKaRa LA Taq™ kit (Takara Bio Europe S. A. S., Saint-Germain-en-Laye, France). Extraction blanks (processed in parallel with the samples) and negative-template PCR controls (NTC) were included for the 16S rRNA gene amplification.

**Table 2:** Primer sequences used for amplification of the 16S rRNA gene.

Primers	Sequence	Length of PCR product (bp)	References
27F	5´-AGAGTTTGATCMTGGCTCAG-3´	1,463	Lane (30)
1492R	5´-GGTTACCTTGTTACGACTT-3´		

The forward primer 27F and reverse primer 1492R were selected based on Lane (30) to amplify a ~ 1,463 bp fragment, covering nearly the entire bacterial 16S rRNA coding region.

Sequencing libraries were prepared using the 16S Barcode kit SQK-RAB204 from Oxford Nanopore Technologies (ONT™, United Kingdom), purified with Agencourt magnetic beads (AMPure XP, Beckman Coulter™), and sequenced using a Flongle MinION flow cell (FLO-FLG001 R10.4.1) on a MinION-Mk1C device (ONT™, United Kingdom) for 24 h.

#### 2.4.2 Data analysis and visualization

FastQ files produced by ONT™ were uploaded to the EPI2ME platform^1^ and analyzed using Fastq 16S 2021.09.09 (Metrichor Agent, ONT™) with the following parameters: quality threshold 10, minimum sequence length 1,400 bases, and BLAST *E*-value of 0.01. Taxonomic assignment was performed using the NCBI_16S database via BLAST, set to default parameters optimized for EPI2ME platform with minimum horizontal coverage of 30% and minimum identity of 77%. Read data were organized using Microsoft Excel platform. Only taxa with relative abundance ≥ 0.1% were considered for analysis and displayed as pie charts. Multiple comparisons of bacterial sequence reads among samples were performed using the Kruskal–Wallis test with Dunn’s test as *post hoc*. Additionally, categorical dichotomous data were expressed as counts and percentages and evaluated using Fisher’s exact test. Statistical analyses were performed using the free online software EZR. Statistical significance was set at *p* < 0.05.

#### 2.4.3 Statistical analysis and calculation of diversity indices

Statistical analyses were conducted using R v.4.1.3 and the “vegan” package.^2^ Alpha diversity per sample was assessed using Shannon’s index, while biodiversity was evaluated using the Menhinick richness index. Shapiro–Wilk test was used to assess the normality of data distribution. A non-paired Wilcoxon rank-sum test (Mann–Whitney *U* test) was applied to compare alpha diversity and biodiversity values. To identify potential sample stratification, beta diversity was assessed using Bray–Curtis dissimilarity matrix calculated from the normalized community data for each category pair. Permutational Multivariate Analysis of Variance (PERMANOVA) test was used to test significant differences in microbial community composition among the defined groups. Following, pairwise *post hoc* tests were performed (RVAideMemoire package), with *p*-values adjusted for multiple comparisons using the Benjamini–Hochberg (BH) method. Statistical significance was set at *p* < 0.05.

## 3 Results

**Figure 1:**
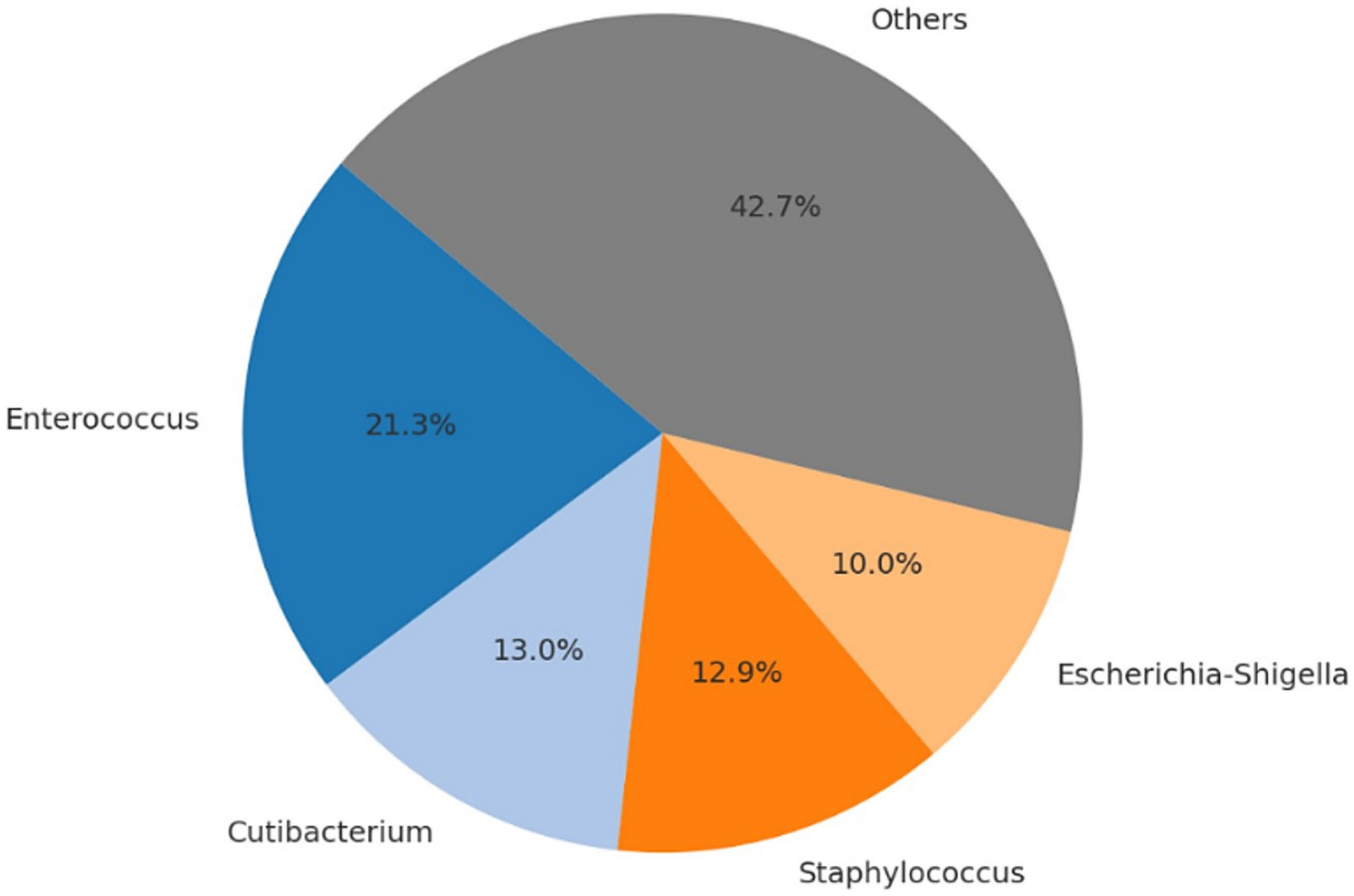
Relative distribution of bacterial genera in oral swabs from newborn puppies (T0). The most abundant genera are *Enterococcus* (21.3%), *Cutibacterium* (13.0%), *Staphylococcus* (12.9%), and *Escherichia-Shigella* (10.0%). All other genera with <5% relative abundance are grouped under “Others” (42.7%). Percentages are based on total sequencing reads.

**Figure 2:**
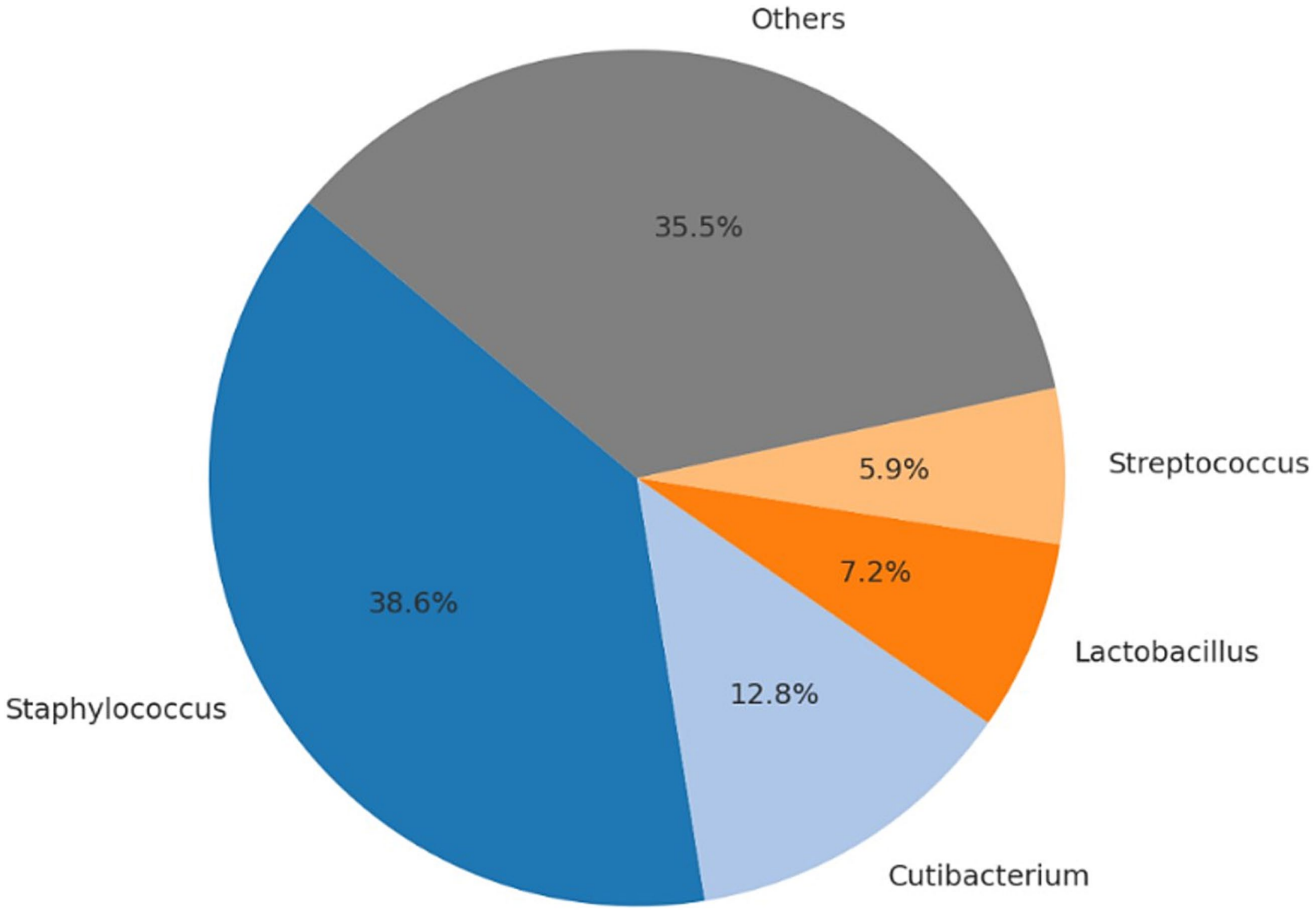
Relative distribution of bacterial genera in oral swabs from puppies at 15 days of age. The most abundant genera are *Staphylococcus* (38.6%), *Cutibacterium* (12.8%), *Lactobacillus* (7.2%) and *Streptococcus* (5.9%). Genera below 5% abundance are aggregated in the “Others” category (35.5%).

**Figure 3:**
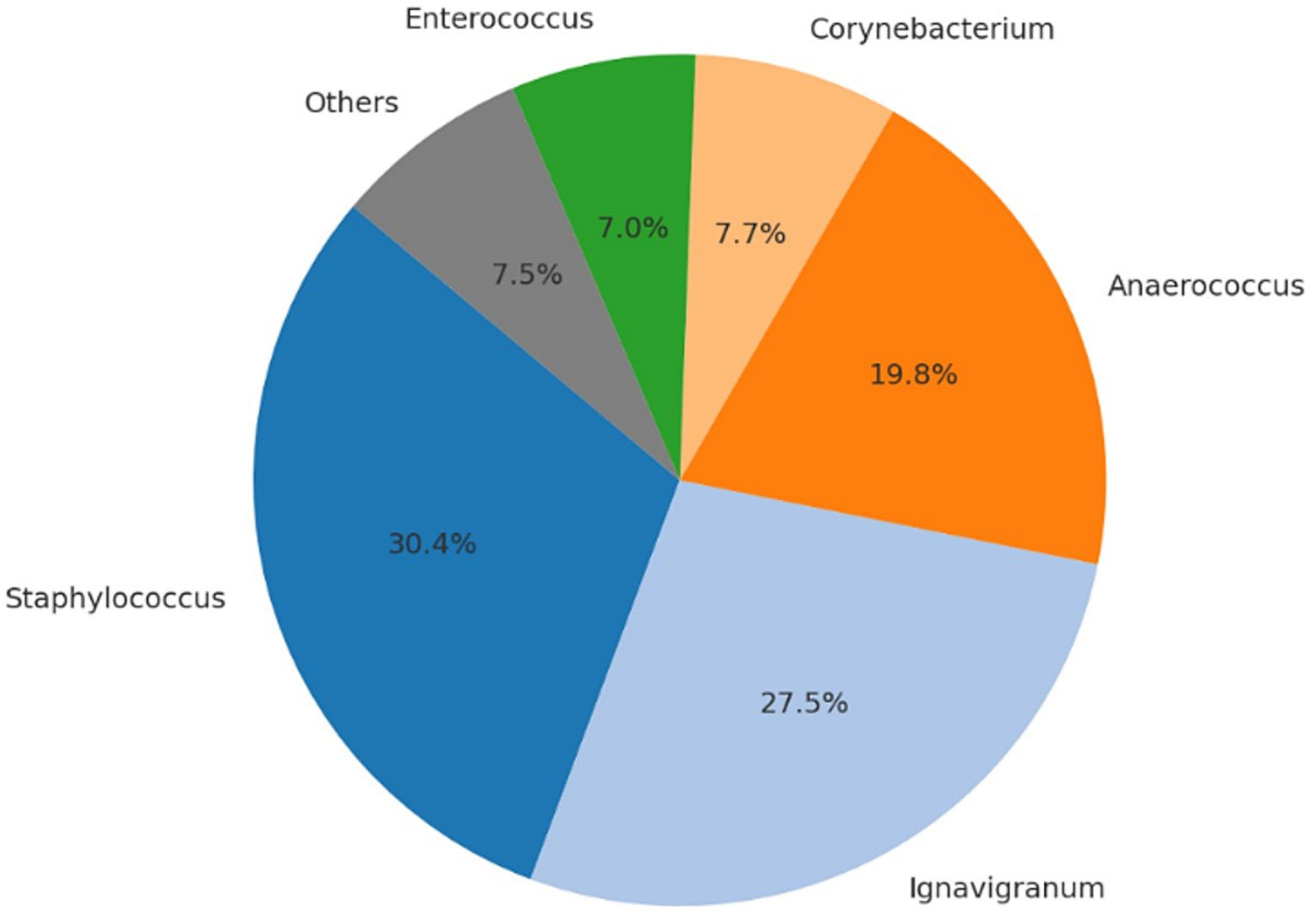
Relative distribution of bacterial genera in maternal colostrum. Predominant genera include *Staphylococcus* (30.4%), *Ignavigranum* (27.5%), *Anaerococcus* (19.8%), *Corynebacterium* (7.7%), and *Enterococcus* (7.0%). The remaining genera (<5%) are grouped under “Others” (7.6%).

**Figure 4:**
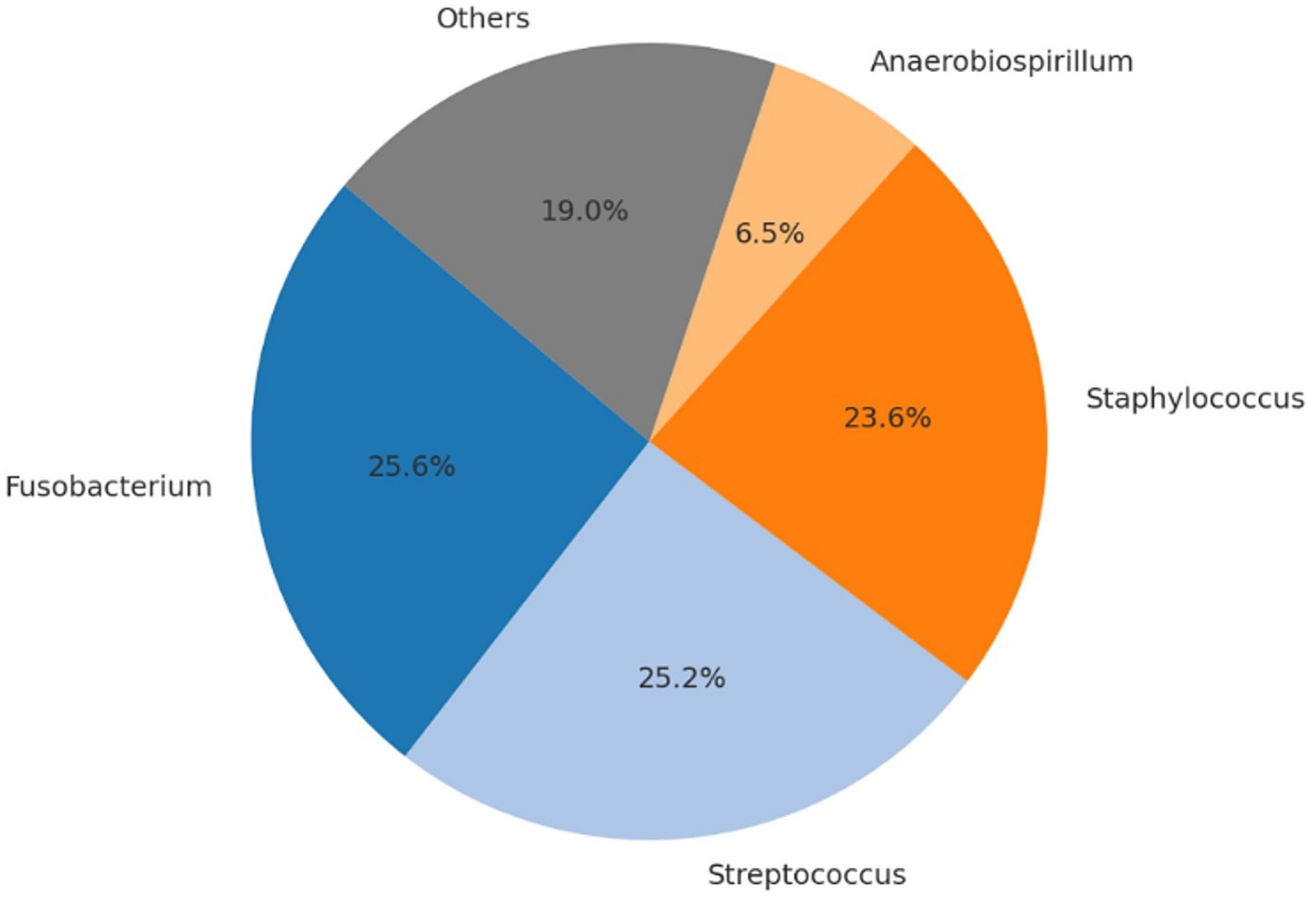
Relative distribution of bacterial genera in the dam oral cavity. Dominant genera are *Fusobacterium* (25.6%), *Streptococcus* (25.2%), *Staphylococcus* (23.6%), *Anaerobiospirillum* (6.5%), and *Megamonas* (2.5%). Genera with relative abundance <5% are grouped under “Others” (16.6%).

No intraoperative or postoperative complications related to either the surgical procedure or anesthesia were recorded during or following cesarean section. All neonates were delivered alive and exhibited normal vitality parameters at birth. No congenital anomalies were observed, and all puppies presented APGAR scores equal to or greater than 7. Following anesthetic recovery, all dams showed no clinical signs of postoperative pain, initiated maternal behavior promptly, and neonates spontaneously began suckling without assistance. Microbial community profiling across the different sampling time points and biological matrices revealed significant shifts in the relative abundance of bacterial taxa. Tables 3–6, Figures 1–4, and Supplementary Tables 1–4 illustrate the percentage distribution of bacterial genera exceeding 5% relative abundance, along with the residual category “Others,” for each sample type. At time point T0 (birth), the oral microbiota of the neonates was characterized by a predominance of *Enterococcus* (21.3%), *Cutibacterium* (13.0%), *Staphylococcus* (12.9%), and *Escherichia-Shigella* (10.0%). The remaining taxa, grouped under the category “Others,” accounted for 42.7% of the total microbial community (Table 3; Figure 1; Supplementary Table 1). After 15 days (T15), a marked predominance of *Staphylococcus* (38.6%) was observed, followed by *Cutibacterium* (12.8%), *Lactobacillus* (7.2%), and *Streptococcus* (5.9%), with a residual proportion of “Others” amounting to 35.5% (Table 4; Figure 2; Supplementary Table 2). In the colostrum sample, the most represented genera were *Staphylococcus* (30.4%), *Ignavigranum* (27.5%), and *Anaerococcus* (19.8%), followed by *Corynebacterium* (7.7%) and *Enterococcus* (7.0%); the residual component accounted for 7.5% (Table 5; Figure 3; Supplementary Table 3). The maternal sample showed a distribution across *Fusobacterium* (25.6%), *Streptococcus* (25.2%), and *Staphylococcus* (23.6%), along with *Anaerobiospirillum* (6.5%); the “Others” category accounted for 19.04% (Table 6; Figure 4; Supplementary Table 4). Alpha diversity among samples, calculated using the Shannon index, ranged from 0.0611 to 0.941 (mean: 0.640; median: 0.621), while biodiversity assessed using the Menhinick Richness index ranged from 0.014 to 0.256 (mean: 0.083; median: 0.063). Alpha diversity, calculated using the Shannon index, showed no significant difference between the T0 and the T15 samples (Wilcoxon rank-sum test, *W* = 105, *p* = 0.855). This suggests that while the community composition may change, the overall richness and evenness of the oral microbiota did not significantly increase or decrease in the first 15 days of life. Beta diversity analysis, which assesses community composition, revealed significant differences. The global PERMANOVA test was highly significant (*p* = 0.0001), indicating that at least one group was different from the others. The “Group” variable (T0, T15, Mother, Colostrum) explained 39.7% of the total variance in the dataset (*R*^2^ = 0.397).

**Table 3:** Number of sequencing reads and relative abundance (%) of bacterial genera identified in oral swabs from newborn puppies (T0).

Genera	Read_count	Abundance_percent (%)
*Enterococcus*	54,736	21.35
*Cutibacterium*	33,438	13.04
*Staphylococcus*	33,050	12.89
*Escherichia-Shigella*	25,758	10.05
Others	109,398	42.67

The “Read Count” column shows the absolute number of sequencing reads assigned to each genera. The “Abundance Percent (%)” column reports the percentage calculated over the total number of reads. Bacterial genera with a relative abundance below 5% have been grouped under the “Others” category.

**Table 4:** Number of sequencing reads and relative abundance (%) of bacterial genera identified in oral swabs from puppies at 15 days of age (Pups Day 15).

Genera	Read_count	Abundance_percent %
*Staphylococcus*	644,454	38.64
*Cutibacterium*	213,151	12.78
*Lactobacillus*	120,092	7.20
*Streptococcus*	97,777	5.86
Others	592,108	35.50

The “Read Count” column indicates the absolute number of sequencing reads assigned to each genera. The “Abundance Percent (%)” column represents the percentage relative to the total reads. Genera with <5% abundance were grouped into the “Others” category.

**Table 5:** Number of sequencing reads and relative abundance (%) of bacterial genera identified in maternal colostrum.

Genera	Read_count	Abundance_percent%
*Staphylococcus*	6,954	30.4226
*Ignavigranum*	6,292	27.5264
*Anaerococcus*	4,532	19.8267
*Corynebacterium*	1,770	7.7434
*Enterococcus*	1,604	7.0172
Others	1,706	7.4635

The “Read Count” column shows the absolute number of reads per genera, while the “Abundance Percent (%)” column expresses the proportion of each genera relative to the total reads. Genera contributing less than 5% were grouped as “Others”.

**Table 6:** Number of sequencing reads and relative abundance (%) of bacterial genera identified in the maternal oral sample (Mother).

Genera	Read count	Abundance percent %
*Fusobacterium*	24,634	25.60
*Streptococcus*	24,284	25.24
*Staphylococcus*	22,744	23.64
*Anaerobiospirillum*	6,242	6.49
Others	18,316	19.04

The “Read Count” column lists the absolute number of sequencing reads assigned to each genera. The “Abundance Percent (%)” column indicates the percentage calculated from the total read count. Genera with a relative abundance lower than 5% were grouped under the “Others” category.

The oral microbiota of puppies at birth (T0) was significantly different from both their 15-day-old (T15) counterparts (*p* = 0.006) and their mothers (*p* = 0.018) (Table 7). However, by day 15, the puppy microbiota (T15) was no longer statistically different from that of the mothers (*p* = 0.192). This indicates a significant shift in community composition during the first 15 days of life, culminating in a microbial profile that converges with and becomes indistinguishable from the maternal oral microbiota. No significant differences were found involving the colostrum samples.

**Table 7:** Results of the pairwise PERMANOVA (*post hoc*) tests for beta diversity (Bray–Curtis dissimilarity) among experimental groups.

(A) Global model results (model: dis ~ group, strata by dam)					
Source	Df	SumofSqs	*R* ^2^	*F*	pr(>*F*)
Model (group)	3	43.638	0.397	6.79	0.0001***
Residual	31	66.366	0.603		
Total	34	110.004	1.000		

(B) Pairwise *post hoc* comparisons (*p*-values adjusted with BH method)	
Groups comparison	*p*-value (BH-adjusted)
T0 vs. T15	0.006**
T0 vs. Mother	0.018*
T0 vs. Colostrum	0.192
T15 vs. Mother	0.192
T15 vs. Colostrum	0.136
Mother vs. Colostrum	0.667

Values shown are *p*-values adjusted using the Benjamini–Hochberg (BH) method. Asterisks indicate statistical significance: *p < 0.05 **p < 0.01, ***p < 0.001.

## 4 Discussion

The microbiota is defined as a dynamic, balanced community of commensal, symbiotic, and potentially pathogenic microorganisms that stably colonize the body surfaces of animals, interacting at various levels with the physiological functions of the host organism (2). The study of microbial community biodiversity, in terms of analysis of richness, composition, and distribution, is a fundamental tool for understanding the ecological structure and interactions among the species present, as well as bacterial relationships with the host (17). This study aimed to assess the temporal variation of the oral microbiota in cesarean-delivered puppies at birth (T0), and at 15 days of age (T15), in comparison with the maternal sources such as colostrum and dam’s oral microbiota. The goal was to describe potential sources of early microbial colonization and to analyze the dynamic changes that the microbiota undergoes over time. The results revealed a high microbial richness index in samples collected from puppies at birth (T0), characterized by pronounced heterogeneity and predominance of the genera *Enterococcus*, *Cutibacterium*, *Staphylococcus*, and *Escherichia-Shigella*. These findings are consistent with the only study currently available that investigates microbial communities in the oral cavity of puppies delivered by cesarean section (14). However, it is important to note the methodological differences: while Kačírová et al. (14) used culture-based isolation techniques, the present study was based on a biomolecular approach for the detection and relative quantification of bacterial sequences. Of particular interest is the highly significant difference observed between the oral microbiota of puppies at T0 and that of their mothers (*p* = 0.018). This result contrasts with findings in human medicine, where several studies have reported a high degree of similarity between the oral microbial profiles of mothers and their newborns, even in cesarean deliveries (18). This discrepancy raises the possibility of additional sources of early colonization, such as the placenta, amniotic fluid, or meconium, which may confer a distinct and more homogeneous microbial imprint among individuals within the same litter. At T15 there was a progressive increase in the relative abundance of *Staphylococcus*, *Lactobacillus*, and *Streptococcus*, accompanied by a concurrent reduction in *Enterococcus* and *Escherichia-Shigella*. Statistical analyses confirmed a significant difference between the two time points analyzed (T0 vs. T15; *p* = 0.006). In contrast, no statistically significant differences were observed between the T15 samples and those from the mothers. This is likely attributable to a combination of behavioral, immunological, environmental, and potentially genetic factors contributing to the progressive maturation and stabilization of the neonatal microbial community. From a behavioral perspective, maternal care in the early postnatal period, particularly licking and grooming, may play a key role, as these represent primary forms of mother-puppy interaction (19). Licking not only stimulates vital neonatal functions but also involves direct and prolonged exposure to maternal oral secretions (20), potentially enabling transmission of microorganisms from the maternal oral cavity to the newborn. Close and repeated physical contact during nursing and rest periods also facilitates continuous bacterial transfer from the mother to the puppies via mucosal and cutaneous surfaces (21). Additional opportunities for microbial exchange may arise from the shared environment, which serves as a common microbial reservoir. The cohabitation of mothers and puppies in the same space, with surfaces and objects contaminated by maternal flora, can facilitate colonization by similar microorganisms (22, 23). A contribution from the host’s genetic and immunological profile cannot be excluded, as it may influence the selection and maintenance of specific bacterial communities through molecular patterns expressed as early as fetal life (24). In this context, the innate immune system of the puppy, still immature but functionally active (25), may act as a selective filter, promoting the growth of certain microorganisms typically present in the maternal oral cavity while inhibiting others (26). These results suggest that, even within the first 2 weeks of life, the oral microbiota of puppies begins to converge toward a compositional profile more closely resembling that of adults. This trend indicates an ecological transition toward a more stable microbial configuration that is better adapted to the specific anatomical site (27). The comparison with maternal colostrum highlighted the presence of *Staphylococcus*, *Enterococcus*, and *Corynebacterium* as shared components, supporting the hypothesis of vertical microbial transmission through breastfeeding (28, 29). However, the lower diversity observed in the colostrum microbiota compared to the oral microbiota of the puppies, both at birth and at 15 days, suggests that environmental exposure and direct maternal contact play a synergistic and likely predominant role in enriching the neonatal microbial community. One of the main strengths of this study lies in the controlled management of experimental conditions. All enrolled subjects were selected from the same breeding facility and were uniformly handled by the same owner, ensuring strict control over environmental and husbandry variables. Similarly, puppies were housed in identical enclosures for each litter, with limited access to a single caretaker responsible for their care and management. This helped reduce potential biases, improve sampling standardization, minimize the risk of contamination, and achieve more reliable and representative results. It is important to disclose some limitations of the present study that could be addressed in future research.

This study has several limitations. First, the small sample size limits the generalizability of our findings; future research with larger cohorts is needed to confirm these observations. Second, a scarcity of literature on the neonatal oral microbiota of puppies, particularly those delivered by cesarean section, made it difficult to compare our results with previous studies. Finally, while next-generation sequencing (NGS) offers high sensitivity, it is susceptible to methodological biases from DNA extraction, amplification, or bioinformatic analysis, which could lead to the over- or underestimation of specific bacterial taxa.

In light of these considerations, future research could focus on expanding the sample size and including a wider range of environmental and biological variables. In particular, it would be valuable to evaluate the microbiota of the placenta, amniotic fluid, the immediate postnatal environment, and the skin and oral microbiota of human caretakers in contact with the puppies. Such investigations would allow for a more precise definition of early microbial colonization sources and their implications for animal health and welfare, within an integrated One Health and preventive medicine framework.

## 5 Conclusion

This study described the early stages of oral microbial colonization in cesarean-delivered canine puppies. It also highlighted the crucial influence exerted by direct contact with the mother and the environment in shaping the composition of the neonatal microbiota. This investigation provides evidence of oral microbiota colonization already present at birth, contributing to the ongoing debate surrounding the validity of the so-called “sterile womb” paradigm. The results demonstrated that the composition of the oral microbiota in puppies evolves significantly during the first 15 days of life. Initially characterized by high heterogeneity and the predominance of opportunistic genera, the microbial profile progressively transitions toward a more stable community structure, increasingly resembling that of the mother. These findings underscore the importance of monitoring microbiota composition during the early stages of development and of considering potential intervention strategies aimed at supporting physiological bacterial colonization, an especially relevant aspect in neonates exposed to factors that predispose to dysbiosis. Finally, this work contributes to expanding current knowledge on the dynamics of vertical microbial transmission in dogs, and it lays the groundwork for future studies aimed at elucidating the functional role of different microbial populations in maintaining both oral and systemic health in puppies. The results also hold broader significance, aligning with a One Health perspective by demonstrating how understanding mother–offspring microbial interactions can provide valuable insights not only for veterinary medicine but also for the study and prevention of microbiota-related disorders in humans.

## Data Availability

The original datasets generated for this study are available in a publicly accessible repository. These data can be found in the NCBI database under the BioProject: PRJNA1368794.

## Footnotes

1
https://epi2me.nanoporetech.com/

2
https://vegandevs.github.io/vegan/
