# Supplementary material for: Oral microbiota in cesarean-delivered puppies
Source: Front Vet Sci. 2025 Dec 8;12:1711728. doi: 10.3389/fvets.2025.1711728 (PMC12719267; doi:10.3389/fvets.2025.1711728)
Supplement: Supplementary file 1 [file Table_1.pdf]

Puppies (0gg)			
**Assigned Taxon**	**NbReads**	**AssignedRank**	**%**
Enterococcus	6951472	Genus	21,35%
Cutibacterium	4246626	Genus	13,04%
Staphylococcus	4197350	Genus	12,89%
Escherichia-Shigella	3271266	Genus	10,05%
Others	13893546	Genus	42,67%

Supplementary Table 1: Bacterial genera detected in newborn puppies at birth (T0), with total reads assigned, taxonomic rank, and relative abundance. “Others” includes genera with low abundance not listed individually.
