# Supplementary material for: Oral microbiota in cesarean-delivered puppies
Source: Front Vet Sci. 2025 Dec 8;12:1711728. doi: 10.3389/fvets.2025.1711728 (PMC12719267; doi:10.3389/fvets.2025.1711728)
Supplement: Supplementary file 2 [file Table_2.pdf]

Puppies (15gg)			
**Assigned Taxon**	**NbReads**	**AssignedRank**	**%**
Staphylococcus	21266982	Genus	38,65%
Cutibacterium	7033983	Genus	12,78%
Lactobacillus	3963036	Genus	7,20%
Streptococcus	3226641	Genus	5,86%
Others	19539564	Genus	35,51%

Supplementary Table 2: Bacterial genera detected in oral swabs from puppies at 15 days of age (T15), with total reads assigned, taxonomic rank, and relative abundance. "Others" includes genera with low abundance not listed individually.
