# Supplementary material for: Oral microbiota in cesarean-delivered puppies
Source: Front Vet Sci. 2025 Dec 8;12:1711728. doi: 10.3389/fvets.2025.1711728 (PMC12719267; doi:10.3389/fvets.2025.1711728)
Supplement: Supplementary file 3 [file Table_3.pdf]

Colostrum			
**Assigned Taxon**	**NbReads**	**AssignedRank**	**%**
Staphylococcus	1787178	Genus	30,42%
Ignavigranum	1617044	Genus	27,52%
Anaerococcus	1164724	Genus	19,83%
Corynebacterium	454890	Genus	7,74%
Enterococcus	412228	Genus	7,02%
Others	438956	Genus	7,47%

Supplementary Table 3: Bacterial genera detected in maternal colostrum, with total reads assigned, taxonomic rank, and relative abundance. “Others” includes genera with low abundance not listed individually.
