# Supplementary material for: Oral microbiota in cesarean-delivered puppies
Source: Front Vet Sci. 2025 Dec 8;12:1711728. doi: 10.3389/fvets.2025.1711728 (PMC12719267; doi:10.3389/fvets.2025.1711728)
Supplement: Supplementary file 4 [file Table_4.pdf]

Mothers			
**Assigned Taxon**	**NbReads**	**AssignedRank**	**%**
Fusobacterium	3645832	Genus	25,60%
Streptococcus	3594032	Genus	25,24%
Staphylococcus	3366112	Genus	23,64%
Anaerobiospirillum	923816	Genus	6,49%
Others	2711952	Genus	19,04%

Supplementary Table 4: Bacterial genera detected in dams oral cavity, with total reads assigned, taxonomic rank, and relative abundance. "Others" includes genera with low abundance not listed individually.
